# ACC/AHA Hypertension Guidelines and CHA_2_DS_2_-VASc Up-Scoring in Patients With Atrial Fibrillation

**DOI:** 10.1001/jamanetworkopen.2023.35722

**Published:** 2023-09-26

**Authors:** Krishna Pundi, Kensey L. Gosch, Alexander C. Perino, Philip G. Jones, Nihar R. Desai, Thomas M. Maddox, Mintu Turakhia

**Affiliations:** 1 Department of Medicine, Stanford University School of Medicine, Stanford, California; 2 St Luke’s Mid America Heart Institute, Kansas City, Missouri; 3 Section of Cardiovascular Medicine, Yale School of Medicine, New Haven, Connecticut; 4 Division of Cardiology, Washington University School of Medicine, St Louis, Missouri

## Abstract

This cohort study compares rates of hypertension among nonhypertensive patients with atrial fibrillation using JNC 8 vs ACC/AHA thresholds.

## Introduction

The CHA_2_DS_2_-VASc score identifies patients with atrial fibrillation (AF) who are likely to derive net clinical benefit from oral anticoagulation (OAC).^1^ One component of CHA_2_DS_2_-VASc is hypertension, defined by contemporary clinical practice.^1^ The 2017 American College of Cardiology/American Heart Association (ACC/AHA) hypertension guidelines lowered the blood pressure (BP) threshold to diagnose hypertension from 140/90 to 130/80 mm Hg.^2^ Among nonhypertensive patients with AF, the new definition may result in earlier hypertension diagnoses, CHA_2_DS_2_-VASc up-scoring, and earlier OAC indications in those with previous scores of 0 or 1.

## Methods

We performed a retrospective cohort study of nonhypertensive patients with AF using the National Cardiovascular Data Registry Practice Innovation and Clinical Excellence (PINNACLE) outpatient quality improvement registry (cohort selection diagram in eFigure in Supplement 1).^3^ The first encounter on or after January 1, 2016, was the index encounter. Informed consent waiver and study authorization were granted by Advarra. This study follows the Strengthening the Reporting of Observational Studies in Epidemiology (STROBE) reporting guideline.

We used 2 hypertension definitions: Eighth Joint National Committee (JNC 8) hypertension, defined as having 2 or more BP measurements with a systolic pressure of 140 mm Hg or higher and/or diastolic pressure of 90 mm Hg or higher within a 2-year span; or 2017 ACC/AHA hypertension, using BP cutoffs of 130 and 80 mm Hg, correspondingly. The primary outcome was CHA_2_DS_2_-VASc up-scoring (details of CHA_2_DS_2_-VASc calculation in eTable in Supplement 1), defined as receiving 1 point for hypertension with 2017 ACC/AHA hypertension but not JNC 8 hypertension, in patients with an initial score of 0 or 1. We used Kaplan-Meier product limit survival estimates to calculate the duration from the first encounter with CHA_2_DS_2_-VASc up-scoring until (1) JNC 8 hypertension diagnosis, (2) CHA_2_DS_2_-VASc score of 2 or higher from accumulating different comorbidities or increase in age, or (3) end of follow-up.

## Results

The analysis cohort comprised 316 388 patients (mean [SD] age, 68 [15] years; 42% female) with AF and outpatient encounters on or after January 1, 2016 (Table). At the index encounter, 53 920 patients (17.0%) met the 2017 ACC/AHA hypertension definition based on previous BP values, of whom 6420 (11.9%) had an index CHA_2_DS_2_-VASc score of 0 and 11 451 (21.2%) had a score of 1 (prior to new hypertension diagnosis). At 36 months, 83.5% of patients met the 2017 ACC/AHA hypertension definition, compared with 53.3% with the JNC 8 hypertension definition (Figure). At 36 months, 51.4% of patients with an index CHA_2_DS_2_-VASc score of 0 had JNC 8 hypertension compared with 86.6% with 2017 ACC/AHA hypertension; in patients with an index CHA_2_DS_2_-VASc score of 1, 50.0% had JNC 8 hypertension compared with 83.0% with 2017 ACC/AHA hypertension.

**Table.** Baseline Characteristics of Nonhypertensive Patients With AF With Outpatient Encounters On or After January 1, 2016

Characteristic	Patients, No. (%) (N = 316 388)
Age, mean (SD), y	68.0 (14.5)
Sex	
Female	134 055 (42.4)
Male	182 333 (57.6)
Race^a^	
Black/African American	9086 (4.3)
White	197 100 (93.0)
Other^b^	5554 (2.6)
Multiracial	192 (0.1)
Insurance	
None	1774 (0.7)
Private	176 120 (71.8)
Medicare	59 320 (24.2)
Medicaid	5794 (2.4)
Other	2390 (1.0)
Height, mean (SD), cm	172.4 (11.0)
Weight, mean (SD), kg	86.1 (23.1)
BMI, mean (SD)	28.9 (6.8)
Heart rate, mean (SD), beats/min	74.0 (15.4)
Blood pressure, mean (SD), mm Hg	
Systolic	122.6 (15.5)
Diastolic	73.1 (10.1)
Diabetes	33 892 (10.7)
Dyslipidemia	96 942 (30.6)
Heart failure	69 620 (22.0)
Stroke/TIA	36 845 (11.6)
Coronary artery disease	90 289 (28.5)
Peripheral vascular disease	21 079 (6.7)
Stroke	28 471 (9.0)
Myocardial infarction	15 157 (4.8)

Abbreviations: AF, atrial fibrillation; BMI, calculated as weight in kilograms divided by height in meters squared; TIA, transient ischemic attack.

^a^ Self-reported.

^b^ Includes American Indian or Alaska Native and Asian.

**Figure.**
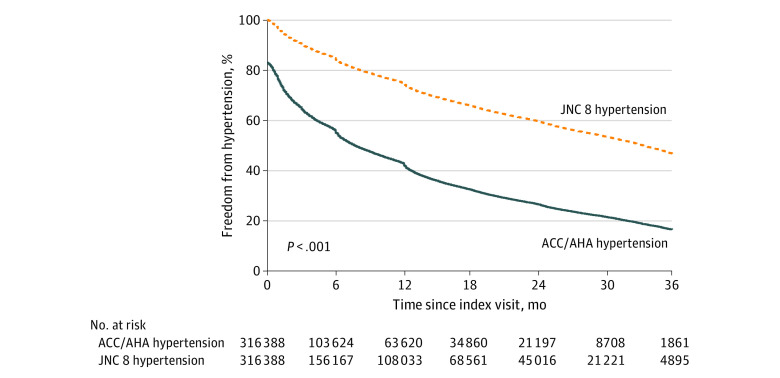
Kaplan-Meier Estimates of Freedom From New Hypertension Diagnoses by JNC 8 and 2017 ACC/AHA Definitions At the index encounter, 53 920 patients (17.0%) met the definition for 2017 American College of Cardiology/American Heart Association (ACC/AHA) hypertension. At 36 months, 83.5% of patients met 2017 ACC/AHA hypertension compared with 53.2% with Eighth Report of the Joint National Committee (JNC 8) hypertension.

In the 113 359 patients with a CHA_2_DS_2_-VASc score of 0 or 1 at the index encounter, 63.1% had up-scoring by 36 months. The mean (SD) duration of up-scoring was 23 (0.2) months and 20 (0.1) months in those with an index score of 0 and 1, accordingly. In up-scored patients with an index score of 0 or 1, 48% and 52% were receiving OAC within 12 months of up-scoring, accordingly.

## Discussion

While the definition of hypertension has changed in response to landmark clinical trials,^4^ CHA_2_DS_2_-VASc was validated using an older hypertension definition^1^ with limited ambulatory BP monitoring and higher BP goals for treatment. Now, patients with AF will meet the CHA_2_DS_2_-VASc threshold for OAC earlier in their disease course. However, it is not known if patients with scores of 1 or 2 using the new hypertension definition have sufficient stroke risk to offset the bleeding risk of OAC and will receive net clinical benefit.^5^

Our study has limitations. PINNACLE cannot incorporate home BP measurements or those from unlinked health care systems. PINNACLE is also an outpatient quality improvement registry not suited to evaluate clinical end points. Our findings support the need to recalibrate AF stroke risk prediction to provide a stronger, contemporary evidence base and incorporate other comorbidities, AF severity and characteristics, and imaging features^6^ to facilitate nuanced patient counseling and shared decision-making.

## Notes

Supplement 1. **eTable.** CHA2DS2-VASc Score Definitions **eFigure.** Cohort Selection Diagram

Supplement 2.
Data Sharing Statement
