# Supplementary material for: ACC/AHA Hypertension Guidelines and CHA2DS2-VASc Up-Scoring in Patients With Atrial Fibrillation
Source: JAMA Netw Open. 2023 Sep 26;6(9):e2335722. doi: 10.1001/jamanetworkopen.2023.35722 (PMC10523168; doi:10.1001/jamanetworkopen.2023.35722)
Supplement: Supplement 1. — eTable. CHA2DS2-VASc Score Definitions eFigure. Cohort Selection Diagram [file jamanetwopen-e2335722-s001.pdf]

Supplemental Online Content

Pundi K, Gosch KL, Perino AC, et al. ACC/AHA hypertension guidelines and CHA_2DS_2-VASc up-scoring in patients with atrial fibrillation. *JAMA Netw Open.* 2023;6(9):e2335722. doi:10.1001/jamanetworkopen.2023.35722

eTable. CHA2DS2-VASc Score Definitions

eFigure. Cohort Selection Diagram

This supplemental material has been provided by the authors to give readers additional information about their work.

© 2023 Pundi K et al. *JAMA Network Open.*

eTable. CHA_2DS_2-VASc Score Definitions

Component	Definition	Points
**C**ongestive heart failure	Presence of heart failure diagnosis, or LV systolic function is moderately or severely reduced	1
**H**ypertension	Presence of hypertension diagnosis or systolic blood pressure ≥ 140 and/or diastolic blood pressure ≥ 90 at two encounters within a 2-year time frame	1
**A**ge	Age at encounter	1-2
**D**iabetes mellitus	Presence of diabetes mellitus diagnosis	1
**S**troke/transient ischemic attack	History of stroke (ischemic or hemorrhagic) or transient ischemic attack	2
**V**ascular disease	History of peripheral artery disease, limb ischemia, claudication, peripheral vascular intervention, peripheral vascular disease, myocardial infarction, coronary artery bypass grafts, percutaneous coronary intervention, carotid endarterectomy, coronary artery disease, lower extremity osteomyelitis, foot/leg cellulitis, or peripheral artery bypass	1
**S**ex **C**ategory	Reported sex	1

© 2023 Pundi K et al. *JAMA Network Open.*

eFigure. Cohort Selection Diagram

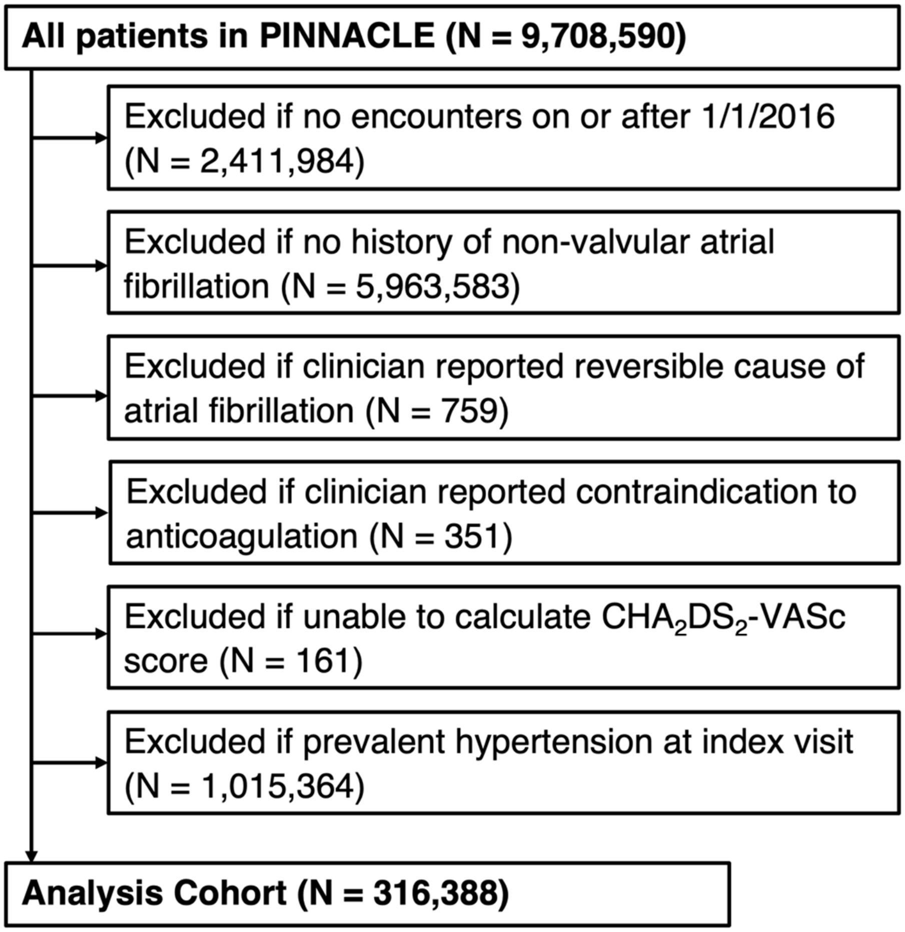

© 2023 Pundi K et al. *JAMA Network Open.*
