# Supplementary material for: ACC/AHA Hypertension Guidelines and CHA2DS2-VASc Up-Scoring in Patients With Atrial Fibrillation
Source: JAMA Netw Open. 2023 Sep 26;6(9):e2335722. doi: 10.1001/jamanetworkopen.2023.35722 (PMC10523168; doi:10.1001/jamanetworkopen.2023.35722)
Supplement: Supplement 2. — Data Sharing Statement [file jamanetwopen-e2335722-s002.pdf]

Data Sharing Statement

Pundi. ACC/AHA Hypertension Guidelines and CHA_2DS_2-VASc Up-Scoring in Patients With Atrial Fibrillation. *JAMA Netw Open*. Published September 26, 2023. doi:10.1001/jamanetworkopen.2023.35722

Data
Data available: No

Additional Information
Explanation for why data not available: Due to data sharing agreements, the investigators of this study cannot make the data freely available. However, qualified investigators can request PINNACLE data from the National Cardiovascular Data Registry, which is managed by the American College of Cardiology (ACC).
